# The race for quantum supremacy: pushing the classical limit for photonic hardware

**DOI:** 10.1093/nsr/nwy125

**Published:** 2018-11-10

**Authors:** Nicolò Spagnolo, Fabio Sciarrino

**Affiliations:** Quantum Lab, Dipartimento di Fisica, Sapienza Università di Roma, Italy

The strong efforts towards the realization of scalable quantum computing are motivated by the promise of super-polynomial speed-up in several computational tasks. A first fundamental step in this route is to provide concrete evidence of ‘quantum supremacy'. Such a regime is obtained when a quantum device is capable of solving a specific problem faster than the best classical hardware. A serious attempt to provide a first demonstration of quantum speed-up with affordable resources is represented by boson sampling [1,2], a non-universal quantum-computational protocol exploiting indistinguishable single photons and linear optical devices. In this context, strong theoretical evidence has been reported that the interference pattern generated by a boson sampler is hard to simulate with classical resources, this pattern being related to the calculation of a hard-to-compute quantity known as a matrix permanent. Formal achievement of the regime of quantum supremacy will answer two very relevant questions: Are we able to benefit from the power of quantum mechanics or does there exist a fundamental reason why this achievement is forbidden? Once this regime has been achieved, are we able to certify the correct functioning of the quantum boson samplers or do we face an undecidability problem?

**Figure 1.**
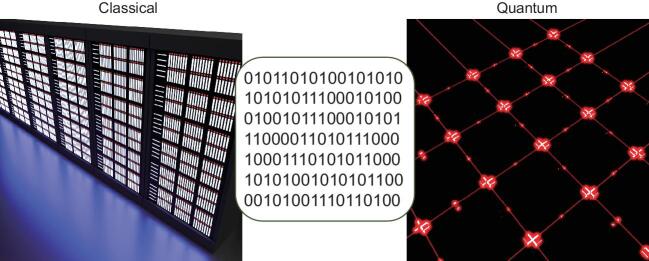
In the race for quantum supremacy, a user who employs the best classical hardware and software, and a second agent who exploits a quantum device compete to solve the boson sampling task faster.

Starting from these intriguing and promising theoretical results, an experimental race kicked off with the aim of reaching the quantum supremacy regime with boson sampling by employing a photonic platform [3]. A large amount of effort has been devoted to pushing the limits of current photonic systems [1], in order to significantly increase the size (number of photons and optical modes) that can be handled with a quantum device. In parallel to this quest for improved quantum performance, it is fundamental to benchmark the limits of a classical simulation of boson sampling. More specifically, in the race for quantum supremacy, what is the best possible classical simulation that can be performed? Such an analysis would be necessary to define the computational capacity possessed by such a classical agent to perform the task (see Fig. 1).

In a recent article, Wu *et al.* [4] have provided a fundamental step to define and push the limits of a classical simulation of boson sampling. The authors have employed Tianhe-2, one of the world's most powerful super-computers, to perform such a task. More specifically, by using such high-performance computing hardware and by employing the fastest algorithm to date, Wu *et al.* have shown that calculating the permanent of a $50 \times 50$ matrix can be performed in 100 min. Recent results [5] have shown that this computation corresponds to the classical hardness of drawing a 50-photon sample from a boson sampling distribution. The strong computational capacity of Tianhe-2 also allowed us to address the precision that the known fastest classical algorithms can reach in such a large-scale calculation. This latter aspect is indeed of fundamental importance, since any reliable classical computation must be accompanied by an appropriate error analysis.

The study reported in Ref. [4] represents by far the largest calculation to date related to the classical simulation of boson sampling, whose relevance can be found in two different but related aspects. First, it raises the bar for any quantum implementation aimed at reaching quantum advantage, and provides a fundamental benchmark to which any photonic hardware must compare. This result suggests that a larger effort must be dedicated to pushing quantum technologies in this direction. Second, improved classical simulation also has important consequences in the context of boson sampling certification, i.e. the verification that a quantum device is correctly performing the required task. Indeed, first validation procedures in the quantum advantage regime must be assisted by powerful classical computational resources that, while requiring longer processing time than quantum hardware, will necessarily have to perform complex calculations.
